# Identifying Residual Psychological Symptoms after Nasal Reconstruction Surgery in Patients with Empty Nose Syndrome

**DOI:** 10.3390/jcm12072635

**Published:** 2023-03-31

**Authors:** Chien-Chia Huang, Pei-Wen Wu, Chi-Che Huang, Po-Hung Chang, Chia-Hsiang Fu, Ta-Jen Lee

**Affiliations:** 1 Division of Rhinology, Department of Otolaryngology, Chang Gung Memorial Hospital, Linkou, Taoyuan City 333, Taiwan; 2 School of Medicine, Chang Gung University, Taoyuan City 333, Taiwan; 3 Department of Otolaryngology, Xiamen Chang Gung Hospital, Xiamen 361000, China

**Keywords:** anxiety, Beck Depression Inventory-II, Beck Anxiety Inventory, empty nose syndrome, depression, nasal reconstruction

## Abstract

Background: Empty nose syndrome (ENS) is a syndrome of paradoxical nasal obstruction that is thought to be mostly caused by inappropriate turbinate procedures. This study aimed to investigate depression- and anxiety-associated psychological symptoms in patients with ENS before and after surgical reconstruction, and to compare them with those of control subjects. Methods: Patients with ENS were prospectively enrolled. The Sino-Nasal Outcome Test-25 (SNOT-25), Empty Nose Syndrome 6-item questionnaire (ENS6Q), Beck Depression Inventory-II (BDI-II), and Beck Anxiety Inventory (BAI) were used to evaluate the participants before and after reconstruction surgery with submucosal Medpor implantation (Stryker, Kalamazoo, MI), as well as control subjects at enrollment. Results: Forty patients with ENS and forty age- and sex-matched controls were recruited. Patients with ENS experienced significant improvement in SNOT-25, ENS6Q, BDI-II, and BAI scores after surgery, but all were significantly greater than those in the control group. Nine patients with ENS (22.5%) had postoperative residual psychological symptoms. Preoperative BDI-II and BAI scores were significant predictors of postoperative residual psychological symptoms. The optimal cut-off value was BDI-II > 28.5 (sensitivity, 77.8%; specificity, 77.4%) in receiver operating characteristic curve analysis. Conclusions: The nasal and psychological evaluations in patients with ENS significantly improved after nasal reconstruction surgery, but both were significantly greater than those in the control group. Identifying individuals who may experience postoperative residual symptoms and providing a multimodal approach, including surgical reconstruction and psychiatric treatment, are suggested.

## 1. Introduction

Empty nose syndrome (ENS) was first described by Eugene Kern and Monika Stenkvist in 1994 as a syndrome of paradoxical nasal obstruction, subjectively speaking, even though a patent nasal airway was observed [1,2]. It is a rare but relevant clinical entity and occurs in about 0.4% surgeries for nasal obstruction, with complete removal of the inferior turbinate being the most common cause of ENS [3]. In addition to the complete resection of the medial wall of maxillary sinus, tumor surgery, oncological procedures, and septum resection, ENS is thought to be mostly caused by previous inappropriate surgery on turbinates. Although the pathophysiology of ENS is not fully understood to date [4,5], disordered recovery in mucosal healing, changes in airflow, and abnormal sensory input from the sense of airflow after nasal surgery may contribute to the development of ENS [4,5,6,7,8,9,10]. The classic symptoms of ENS are nasal obstruction, burning, dryness, crusting, suffocation, and impaired air sensation through the nasal cavity [11,12]. These patients usually have undergone surgery to relieve nasal obstruction, but symptoms deteriorate despite achieving the desired patent anatomic outcome.

Beyond physical symptoms, patients with ENS may carry significant psychological symptoms, including chronic fatigue, frustration, irritability, anger, anxiety, and depression [13,14,15,16]. Using a questionnaire investigation, our previous study showed that 51% of ENS patients experienced comorbid depression and 73% experienced comorbid anxiety [15]. In addition, patients with ENS also experience significantly impaired sleep quality and sleepiness [17]. All of these factors contribute to a negative impact on quality of life and substantial difficulties in many daily activities [13].

Although treatment with surgical reconstruction for patients with ENS is effective in improving both nasal and psychological symptoms, residual disease may be detected in some cases. Furthermore, the severity of preoperative psychological symptoms is an important determining factor for surgical outcomes and residual disease after nasal reconstruction surgery [18]. Suicidal ideation can be identified in up to 37.1% and 6.5% of patients with ENS before and after nasal reconstruction surgery, respectively [19]. Thus, it is important to evaluate psychological symptoms in patients with ENS, even after nasal reconstruction surgery. In this study, we investigated depression- and anxiety-associated psychological symptoms in patients with ENS before and after surgical reconstruction and compared them with those in control subjects. These results will be beneficial for identifying residual psychological symptoms after nasal reconstruction surgery and optimizing patient-centered care for patients with ENS.

## 2. Materials and Methods

### 2.1. Patients

After approval by the institutional review board (IRB numbers: 201601703A3, 201702048B0C501, 201802147A3, and 201902001A3), patients with ENS planning to receive nasal reconstruction by submucosal Medpor implantation (Stryker, Kalamazoo, MI, USA) were prospectively enrolled from 2017 to 2020, after informed consent was obtained. The diagnosis of ENS was based on the symptoms of paradoxical nasal obstruction, history of a previous surgical procedure for inferior turbinate reduction, endoscopic findings of the loss of inferior turbinate tissue and patent nasal airway, and a positive cotton test. A cotton test was performed at enrollment, as described in previous studies [6,12]. In brief, evaluation of nasal symptoms while breathing through the nose before and after the placement of a moistened cotton ball in the widest area of the nasal cavity was performed. Improvements in nasal symptoms after the placement of a moistened cotton ball indicated positive results. The exclusion criteria were as follows: (1) other sinonasal diseases such as rhinosinusitis, neoplasm, or craniofacial anomalies; and (2) psychiatric disorders managed by psychiatrists.

An age- and sex-matched control group was constructed by the enrollment of subjects who visited the otolaryngology outpatient clinic for a problem other than nasal symptoms after nasal evaluation using the nasal symptom questionnaire and endoscopic examination. Informed consent was obtained at enrollment (IRB number: 202201142B0). The demographic data of all participants, including age, sex, and surgical history, were collected for analysis.

### 2.2. Submucosal Medpor Implantation

All patients with ENS received endoscopic-assisted submucosal Medpor implantation, as described previously [14,18]. In brief, the implantation of Medpor in pieces via a submucosal pocket created by an incision on the lateral wall was performed under endoscopic assistance. After surgery, postoperative follow-up at the outpatient clinic depended on the status of mucosal recovery and was usually performed once a week during the first month and then every 3 months after surgery. Two weeks after surgery, ENS patients were instructed to perform nasal douching with warm saline and to use intranasal corticosteroids.

### 2.3. Questionnaire Evaluations

The Chinese versions of the Sino-Nasal Outcome Test-25 (SNOT-25) [15,20], Empty Nose Syndrome 6-item Questionnaire (ENS6Q) [12], Beck Depression Inventory-II (BDI-II) [21] and Beck Anxiety Inventory (BAI) [22] were used to evaluate the severity of ENS symptoms and psychological burden (depression and anxiety) in patients with ENS prior to, 6 months, and 1 year after surgery, as well as in the control group at enrollment.

For the ENS evaluation by SNOT-25 and ENS6Q, participants graded each ENS-related symptom from 0 (no symptoms) to 5 (the most severe) [20]. Scores ranging from 0 to 125 and 0 to 30 were obtained in SNOT-25 and ENS6Q, respectively.

For the psychological burden evaluations by the BDI-II and BAI instruments, participants graded each of the 21 depression- and anxiety-related symptoms from 0 (no symptoms) to 3 (the most severe), with scores ranging from 0 to 63 in both the BDI-II and BAI, respectively [14]. The 95th percentiles of the BDI-II and BAI scores in the control group were calculated. Postoperative BDI-II and BAI scores in ENS patients greater than the 95th percentile of the BDI-II and BAI scores in the control group were defined as indicating residual psychological symptoms.

### 2.4. Statistical Analyses

Data are presented as mean ± standard deviation (SD) and were statistically analyzed using GraphPad Prism 5 (GraphPad Software, San Diego, CA, USA) and SPSS version 26.0 (IBM, Armonk, NY, USA). Categorical variables were compared using the *x*^2^ test or Fisher’s exact test as appropriate. Continuous variables were compared using the Mann–Whitney *U* test, Wilcoxon signed-rank test, or *t*-test between the groups according to the results of the D’Agostino–Pearson omnibus normality test. Univariate and multivariate logistic regression analyses were used to assess predictive variables for postoperative residual psychological symptoms. To identify and characterize the sensitivity and specificity of the predictive variables for identifying ENS patients with postoperative residual psychological symptoms, receiver operating characteristic (ROC) curves were analyzed and the area under the ROC curve (AUC) was calculated. Statistical significance was set at *p* < 0.05.

## 3. Results

### 3.1. Clinical Characteristics of the Participants

Forty patients with ENS, including ten women and thirty men, were enrolled in the study. Forty age- and sex-matched participants were enrolled in the control group. Table 1 summarizes the participants’ demographic data. All patients with ENS had a history of surgery performed on their inferior turbinates. There were 26 (65.0%) and 13 (32.5%) patients with a history of concomitant nasal septal surgery and endoscopic sinus surgery, respectively.

### 3.2. Questionnaire Evaluation

**Figure 1:**
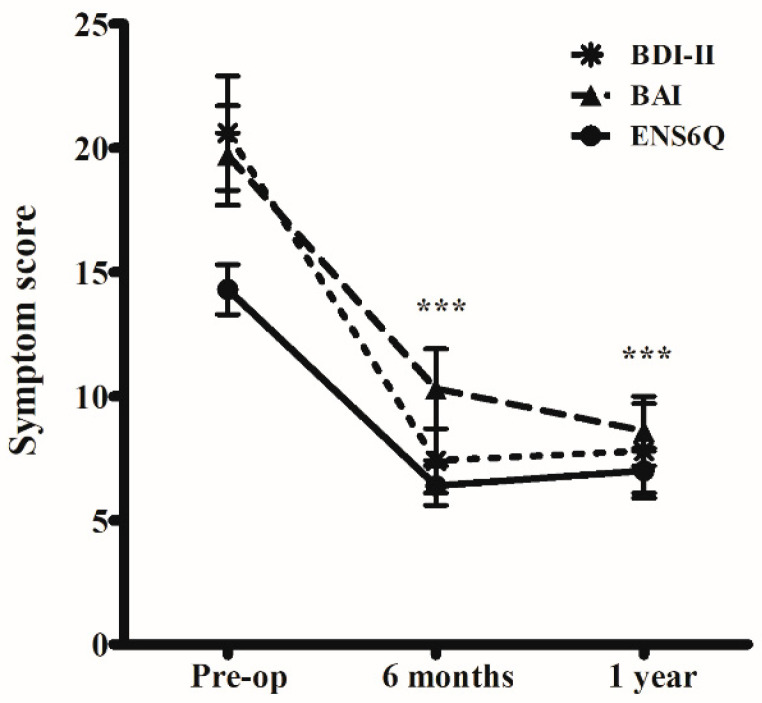
Subjective evaluations of patients with empty nose syndrome (ENS) perioperatively. Surgical reconstruction of nasal cavity by submucosal Medpor implantation (Stryker, Kalamazoo, MI, USA) significantly improved the symptoms of ENS patients evaluated by the Empty Nose Syndrome 6-item Questionnaire (ENS6Q), Beck Depression Inventory-II (BDI-II), and Beck Anxiety Inventory (BAI). *** *p* < 0.001, compared to preoperative (Pre-op) evaluations using the Wilcoxon signed-rank test.

**Figure 2:**
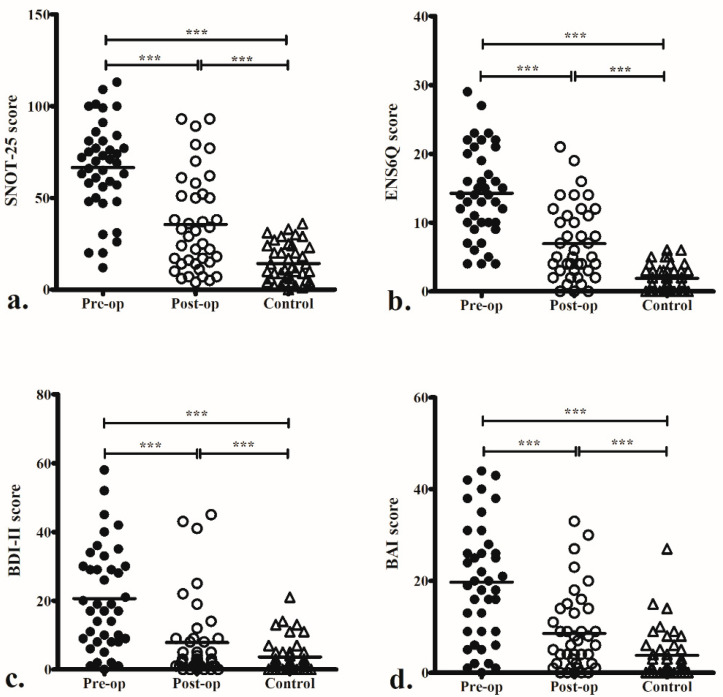
Preoperative (Pre-op) and 1 year postoperative (Post-op) 25-Item Sino-Nasal Outcome Test (SNOT-25) (**a**), Empty Nose Syndrome 6-item Questionnaire (ENS6Q) (**b**), Beck Depression Inventory-II (BDI-II) (**c**), and Beck Anxiety Inventory (BAI) (**d**) in patients with empty nose syndrome and the control group. *** *p* < 0.001.

The ENS6Q, BDI-II, and BAI scores in ENS patients significantly improved 6 months and 1 year after surgery compared to before surgery (Figure 1). There was no difference between these evaluations at 6 months and 1 year postoperatively. Symptom scores evaluated by SNOT-25, ENS6Q, BDI-II, and BAI were all significantly greater in ENS patients than in the control group both before and after surgery (Figure 2).

The 95th percentiles of BDI-II and BAI scores were 13.95 and 14.95, respectively, in the control group. Nine patients with ENS (22.5%) had BDI-II ≥ 14 and/or BAI ≥ 15 at 1 year post operation, and they were considered to have postoperative residual psychological symptoms. Further analysis using regression models showed that the preoperative BDI-II score (odds ratio (OR) = 1.14, *p* = 0.004) and BAI score (OR = 1.08, *p* = 0.033) were significant predictors of postoperative residual psychological symptoms in the univariate analysis, and the preoperative BDI-II score (OR = 1.17, *p* = 0.013) was a significant predictor of postoperative residual psychological symptoms in the multivariate analysis (Table 2).

**Figure 3:**
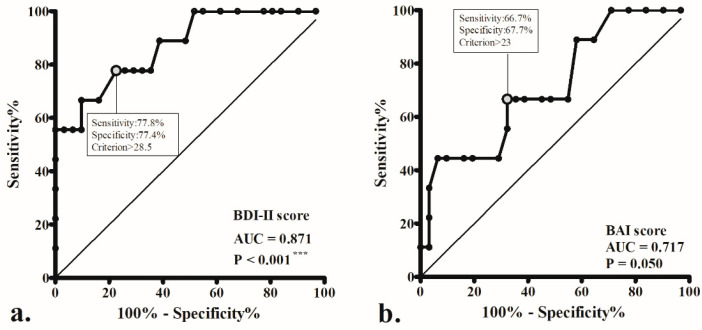
Receiver operating characteristic (ROC) curves to detect empty nose syndrome patients with residual psychological symptoms after surgery (postoperative Beck Depression Inventory II (BDI)-II scores of ≥ 15 and/or Beck Anxiety Inventory (BAI) scores of ≥14), using the variables of the preoperative BDI-II (**a**) and BAI scores (**b**). The optimal cut-offs for these metrics (maximizing the sum of sensitivity and specificity) are indicated. AUC: area under the ROC curve; *** *p* < 0.001.

ROC curves were generated, and the AUC was calculated to evaluate the sensitivity and specificity of the preoperative BDI-II and BAI scores in identifying ENS patients with postoperative residual psychological symptoms after nasal reconstruction surgery (Figure 3). The ROC curves of the BDI-II (AUC = 0.871, *p* < 0.001) had an AUC significantly greater than 0.5 for the prediction of postoperative residual psychological symptoms. The optimal cutoff value (maximizing the sum of sensitivity and specificity) was BDI-II > 28.5 (sensitivity, 77.8%; specificity, 77.4%).

## 4. Discussion

Previous studies have demonstrated that anxiety and depression are prevalent comorbidities in patients with ENS [13,14,15,16]. Surgical reconstruction of the nasal cavity can reduce the airspace as well as the airflow in ENS patients and is effective in improving both nasal and psychological symptoms. In addition, preoperative psychologic evaluations by BDI-II and BAI could be predictors of surgical outcomes and residual disease [18]. The current study revealed that 9 of 40 (22.5%) patients with ENS had a BDI-II ≥ 14 and/or BAI ≥ 15 at 1 year post operation, and they were considered to have postoperative residual psychological symptoms. Nevertheless, Tian et al. reported that patients who have undergone turbinate-sparing procedures but present with ENS symptoms and meet the diagnostic criteria for somatic symptom disorder might benefit from psychiatric treatment, including cognitive–behavioral therapy plus antidepressants for their somatic symptom burden, depression, and anxiety [23]. Thus, we identified ENS patients with residual psychological symptoms after nasal reconstruction surgery in comparison to the control subjects in the current study. These patients may benefit from further psychiatric therapy before or after nasal reconstruction. Furthermore, the preoperative BDI-II and BAI scores were significant predictors of postoperative residual psychological symptoms in regression analysis, and BDI-II > 28.5 was determined as the optimal cut-off value in predicting postoperative residual psychological symptoms (sensitivity, 77.8%; specificity, 77.4%). These results could be beneficial for optimizing patient-centered care for patients with ENS.

ENS is an uncommon and poorly understood complication of turbinate reduction surgery. A recent study using computational fluid dynamic analysis demonstrated that the removal of the inferior turbinate could lead to a significant decrease in airflow in the inferior meatus and an increased airflow around the middle meatus [24,25]. This lack of airflow in the inferior meatus contributes to a reduction in the shear force on the lateral nasal wall, resulting in the diminished stimulation of the mechanoreceptors, which may cause the feeling of suffocation or obstruction in ENS patients [5].

However, it is unknown why a small proportion of patients develop ENS, and why most patients have no such problems after the same turbinate procedures. The extent of the loss of turbinate tissue is not well-correlated with the symptom burden [5]. Residual or persistent disease was found in some cases after nasal reconstruction surgery, especially in those with a greater burden of psychological symptoms [18]. Thus, it is assumed that there might be a psychogenic component associated with these clinical manifestations of ENS. Although poor mental health status in ENS patients has been linked to poor nasal perception and emotional regulation deficits, it is difficult to clarify which disease gives rise to the other [26]. However, the connection between nasal perception and emotional control was investigated in a functional magnetic resonance imaging study that showed the deactivation of emotional processing areas after the successful pseudo-decongestant stimulatory effect of menthol in ENS patients [27]. Taken together, predisposing psychogenic characteristics such as sensitivity to nasal airflow change may increase the risk of developing ENS after alteration in nasal perception due to turbinate surgery. Thus, it is important to evaluate aspects of nasal perception such as postural congestion, response to topical nasal decongestants, and awareness of fluctuating nasal congestion in all turbinate reduction candidates [5].

A previous study by Mangin et al. revealed that hyperventilation syndrome is frequent in patients with ENS [28]. Another study also reported on a cohort of ENS individuals carrying a significant psychological burden and marked difficulties with many activities of daily life, suggesting a multimodal approach that included the surgical construction of tissues and cognitive–behavioral therapy (CBT) to address the psychological issue [13]. Our previous study also demonstrated that suicidal thoughts were frequently identified in patients with ENS [19]. Empty nose syndrome patients with suicidal thoughts experienced significantly more severe symptoms, impaired quality of life, and greater psychological burden than those without suicidal thoughts. Our current study further emphasizes the importance of psychologic evaluation, especially in cases of anxiety and depression, in order to manage these patients with ENS. Perioperative psychologic evaluation could help in predicting surgical outcomes and identifying patients with residual disease. These findings emphasize screening for these conditions and structuring care around both surgery and CBT, according to the patients’ situation, through collaboration with specialists in otolaryngology, psychiatry, and psychology for optimal outcomes.

The SNOT-25 is a modification of the SNOT-20 with five additional ENS-specific symptoms: dryness, difficulty with nasal breathing, suffocation, excessively open nose, and nasal crusting [6]. SNOT-25 comprises the rhinogenic symptom domain, extra-nasal rhinological symptom domain, ear/facial symptom domain, sleep dysfunction domain, psychological dysfunction domain, and empty nose symptom domain [11,15]. Nevertheless, the ENS6Q, developed by Nayak in 2016, is a validated, ENS-specific detection instrument [12]. The ENS6Q contains six items: dryness, lack of air sensation going through the nasal cavities, suffocation, nose feeling too open, nasal crusting, and nasal burning. A measured ENS6Q score ≥ 10.5 suggests the possible presence of ENS, according to a previous report [29]. Both SNOT-25 and ENS6Q were helpful in evaluating perioperative symptoms for patients with ENS. Our previous study revealed that the ENS6Q score had a good correlation with the BDI-II and BAI scores preoperatively, but it was not associated with postoperative BDI-II and BAI scores. Hence, a simultaneous psychological assessment is necessary when evaluating patients using the ENS6Q [20].

BAI and BDI-II are both commonly used self-administered instruments to detect symptoms of anxiety and depression, and to screen patients with possible clinical anxiety and depression [21,22]. BDI was developed by Beck et al. in 1961 and was revised as the BDI-II in 1996. Compared with BDI, the BDI-II showed significant improvement in sensitivity and reliability [21]. Similarly, the BAI was also developed by Beck et al. and is widely used to evaluate anxiety and delineate patients from nonclinical subjects [22]. Our previous study showed that there was a good correlation between the perioperative changes in the BAI and BDI-II scores as well as in the sleep dysfunction and empty nose symptom domains of the SNOT-25. Additionally, psychological evaluation could predict outcomes and detect patients with residual disease after surgical reconstruction [15].

Furthermore, our results in the current study reveal that the preoperative BDI-II and BAI scores were associated with postoperative residual psychological symptoms, and a BDI-II > 28.5 is the optimal cut-off value for prediction. Recognizing individuals who may experience postoperative residual symptoms and providing appropriate preoperative interventions are critical to achieve optimal surgical outcomes. Since patients with ENS usually carry a significant psychological burden that negatively impacts their quality of life and surgical outcome, a comprehensive perioperative psychological evaluation and a multimodal approach, including surgical reconstruction of the nasal cavity and psychiatric treatment to address the psychological issue, are suggested [18].

This study has some limitations that warrant consideration. First, the placebo effect could not be excluded without recruiting a sham surgery group, which is not feasible in a clinical study with ethical considerations. Second, subjects who visited the otolaryngology outpatient clinic for problems other than nasal symptoms were not ideal candidates for the control group. These participants may also carry some degree of psychological burden. However, our results reveal that BDI-II and BAI values were all significantly greater in ENS patients than in the control group, even after surgery. This demonstrates a significant psychological burden of ENS, which would be greater than that of people without ENS. Third, patients with ENS with comorbid psychiatric disorders were excluded because of the difficulty in evaluating nasal symptoms and differentiating the effects of psychiatric therapy and surgery in patients with psychiatric disorders.

## 5. Conclusions

Nasal and psychological evaluations in ENS patients significantly improved after nasal reconstruction surgery, but were still significantly greater than those in the control group. BDI-II > 28.5 is the optimal cut-off value in predicting postoperative residual psychological symptoms. Identifying individuals who may experience postoperative residual psychological symptoms and providing a multimodal approach, including surgical reconstruction and psychiatric treatment, are critical for achieving optimal therapeutic outcomes.

## Figures and Tables

**Table 1:** Clinical characteristics of the study participants.

	ENS	Control	*p* Value ^†^
Case number, n (%)	40	40	
Age (year)	45.0 ± 13.0	44.7 ± 12.6	0.862
Female: male, n	10:30	10:30	1.000
Smoker, n (%)	9 (22.5)	7 (17.5)	0.781
Previous nasal surgery:			
Inferior turbinate surgery, n (%)	40 (100)	0 (0)	
Nasal septal surgery, n (%)	26 (65.0)	0 (0)	
Endoscopic sinus surgery, n (%)	13 (32.5)	0 (0)	
Caldwell–Luc operation, n (%)	2 (5.0)	0 (0)	

Data are represented as mean ± SD. ENS, empty nose syndrome. ^†^ Categorical variables were analyzed using the chi-square test and continuous variables were compared using the Mann–Whitney U test.

**Table 2:** Regression analysis for postoperative residual psychological symptoms.

Variables	Univariate Analysis		Multivariate Analysis	
	Odds Ratio (95% CI)	*p*	Odds Ratio (95% CI)	*p*
Age	0.98 (0.92–1.04)	0.415		
Gender	0.82 (0.14–4.80)	0.827		
Pre-op SNOT-25	1.03 (0.99–1.06)	0.150		
Pre-op ENS6Q	1.10 (0.97–1.25)	0.122		
Pre-op BDI-II	1.14 (1.04–1.25)	0.004 **	1.17 (1.03–1.33)	0.013 *
Pre-op BAI	1.08 (1.01–1.15)	0.033 *	0.96 (0.86–1.08)	0.505

CI, confidence interval; Pre-op, preoperative; SNOT-25, 25-Item Sino-Nasal Outcome Test; ENS6Q, Empty Nose Syndrome 6-item Questionnaire; BDI-II, Beck Depression Inventory II; BAI, Beck Anxiety Inventory. * *p* < 0.05; ** *p* < 0.01.

## Data Availability

All data described in the study are presented in the manuscript. The datasets analyzed are available from the corresponding author on reasonable request.

## References

[1] Scheithauer M.O. (2010). Surgery of the turbinates and “empty nose” syndrome. GMS Curr. Top. Otorhinolaryngol. Head Neck Surg..

[2] Moore E.J., Kern E.B. (2001). Atrophic rhinitis: A review of 242 cases. Am. J. Rhinol..

[3] Shin C.H., Jang Y.J. (2023). Factors affecting the complication rate of septoplasty: Analysis of 1506 consecutive cases of single surgeon. Facial Plast. Surg..

[4] Sozansky J., Houser S.M. (2015). Pathophysiology of empty nose syndrome. Laryngoscope.

[5] Kanjanawasee D., Campbell R.G., Rimmer J., Alvarado R., Kanjanaumporn J., Snidvongs K., Kalish L., Harvey R.J., Sacks R. (2022). Empty Nose Syndrome Pathophysiology: A Systematic Review. Otolaryngol. Head Neck Surg..

[6] Chhabra N., Houser S.M. (2009). The diagnosis and management of empty nose syndrome. Otolaryngol. Clin. N. Am..

[7] Zhao K., Blacker K., Luo Y., Bryant B., Jiang J. (2011). Perceiving nasal patency through mucosal cooling rather than air temperature or nasal resistance. PLoS ONE.

[8] Li C., Farag A.A., Maza G., McGhee S., Ciccone M.A., Deshpande B., Pribitkin E.A., Otto B.A., Zhao K. (2018). Investigation of the abnormal nasal aerodynamics and trigeminal functions among empty nose syndrome patients. Int. Forum Allergy Rhinol..

[9] Konstantinidis I., Tsakiropoulou E., Chatziavramidis A., Ikonomidis C., Markou K. (2017). Intranasal trigeminal function in patients with empty nose syndrome. Laryngoscope.

[10] Wu C.L., Fu C.H., Lee T.J. (2021). Distinct Histopathology Characteristics in Empty Nose Syndrome. Laryngoscope.

[11] Jiang C., Wong F., Chen K., Shi R. (2014). Assessment of surgical results in patients with empty nose syndrome using the 25-item sino-nasal outcome test evaluation. JAMA Otolaryngol. Head Neck Surg..

[12] Velasquez N., Thamboo A., Habib A.R., Huang Z., Nayak J.V. (2017). The Empty Nose Syndrome 6-Item Questionnaire (ENS6Q): A validated 6-item questionnaire as a diagnostic aid for empty nose syndrome patients. Int. Forum Allergy Rhinol..

[13] Manji J., Nayak J.V., Thamboo A. (2018). The functional and psychological burden of empty nose syndrome. Int. Forum Allergy Rhinol..

[14] Lee T.J., Fu C.H., Wu C.L., Tam Y.Y., Huang C.C., Chang P.H., Chen Y.W., Wu M.H. (2016). Evaluation of depression and anxiety in empty nose syndrome after surgical treatment. Laryngoscope.

[15] Huang C.C., Wu P.W., Fu C.H., Huang C.C., Chang P.H., Wu C.L., Lee T.J. (2019). What drives depression in empty nose syndrome? A Sinonasal Outcome Test-25 subdomain analysis. Rhinology.

[16] Lamb M., Bacon D.R., Zeatoun A., Onourah P., Thorp B.D., Abramowitz J., Ebert C.S., Kimple A.J., Senior B.A. (2022). Mental health burden of empty nose syndrome compared to chronic rhinosinusitis and chronic rhinitis. Int. Forum Allergy Rhinol..

[17] Huang C.C., Lee C.C., Wei P.W., Chuang C.C., Lee Y.S., Chang P.H., Huang C.C., Fu C.H., Lee T.J. (2023). Sleep impairment in patients with empty nose syndrome. Rhinology.

[18] Huang C.C., Wu P.W., Fu C.H., Huang C.C., Chang P.H., Lee T.J. (2021). Impact of Psychologic Burden on Surgical Outcome in Empty Nose Syndrome. Laryngoscope.

[19] Huang C.C., Wu P.W., Lee C.C., Chang P.H., Huang C.C., Lee T.J. (2022). Suicidal thoughts in patients with empty nose syndrome. Laryngoscope Investig. Otolaryngol..

[20] Huang C.C., Wu P.W., Lee C.C., Huang C.C., Fu C.H., Chang P.H., Lee T.J. (2022). Comparison of SNOT-25 and ENS6Q in evaluating patients with empty nose syndrome. Laryngoscope Investig. Otolaryngol..

[21] Beck A.T., Steer R.A., Brown G.K. (1996). Manual for the Beck Depression Inventory-II.

[22] Beck A.T., Epstein N., Brown G., Steer R.A. (1988). An inventory for measuring clinical anxiety: Psychometirc properties. J. Consult. Clin. Psychol..

[23] Tian P., Hu J., Ma Y., Zhou C., Liu X., Dang H., Zou H. (2021). The clinical effect of psychosomatic interventions on empty nose syndrome secondary to turbinate-sparing techniques: A prospective self-controlled study. Int. Forum Allergy Rhinol..

[24] Malik J., Dholakia S., Spector B.M., Yang A., Kim D., Borchard N.A., Thamboo A., Zhao K., Nayak J.V. (2021). Inferior meatus augmentation procedure (IMAP) normalizes nasal airflow patterns in empty nose syndrome patients via computational fluid dynamics (CFD) modeling. Int. Forum Allergy Rhinol..

[25] Malik J., Li C., Maza G., Farag A.A., Krebs J.P., McGhee S., Zappitelli G., Deshpande B., Otto B.A., Zhao K. (2019). Computational fluid dynamic analysis of aggressive turbinate reductions: Is it a culprit of empty nose syndrome?. Int. Forum Allergy Rhinol..

[26] Berking M., Wupperman P. (2012). Emotion regulation and mental health: Recent findings, current challenges, and future directions. Curr. Opin. Psychiatry.

[27] Freund W., Wunderlich A.P., Stöcker T., Schmitz B.L., Scheithauer M.O. (2011). Empty nose syndrome: Limbic system activation observed by functional magnetic resonance imaging. Laryngoscope.

[28] Mangin D., Bequignon E., Zerah-Lancner F., Isabey D., Louis B., Adnot S., Papon J.F., Coste A., Boyer L., Devars du Mayne M. (2017). Investigating hyperventilation syndrome in patients suffering from empty nose syndrome. Laryngoscope.

[29] Thamboo A., Velasquez N., Habib A.R., Zarabanda D., Paknezhad H., Nayak J.V. (2017). Defining surgical criteria for empty nose syndrome: Validation of the office-based cotton test and clinical interpretability of the validated Empty Nose Syndrome 6-Item Questionnaire. Laryngoscope.

